# Onsite/offsite social commerce adoption for SMEs using fuzzy linguistic decision making in complex framework

**DOI:** 10.1007/s12652-022-04157-5

**Published:** 2022-07-09

**Authors:** Walayat Hussain, Jose M. Merigo

**Affiliations:** 1 Victoria University Business School, Victoria University, Melbourne, 3000 Australia; 2 Faculty of Engineering and Information Technology, University of Technology Sydney, Ultimo, 2007 Australia

**Keywords:** Social commerce adoption, TOE framework, FAHP, OWA operator, Fuzzy linguistic, Multi-criteria group decision making

## Abstract

There has been a growing social commerce adoption trend among SMEs for few years. However, it is often a challenging strategic task for SMEs to choose the right type of social commerce. SMEs usually have a limited budget, technical skills and resources and want to maximise productivity with those limited resources. There is much literature that discusses the social commerce adoption strategy for SMEs. However, there is no work to enable SMEs to choose social commerce—onsite/offsite or hybrid strategy. Moreover, very few studies allow the decision-makers to handle uncertain, complex nonlinear relationships of social commerce adoption factors. The paper proposes a fuzzy linguistic multi-criteria group decision-making in a complex framework for onsite, offsite social commerce adoption to address the problem. The proposed approach uses a novel hybrid approach by combining FAHP, FOWA and selection criteria of the technological–organisation–environment (TOE) framework. Unlike previous methods, the proposed approach uses the decision maker's attitudinal characteristics and recommends intelligently using the OWA operator. The approach further demonstrates the decision behaviour of the decision-makers with Fuzzy Minimum (FMin), Fuzzy Maximum (FMax), Laplace criteria, Hurwicz criteria, FWA, FOWA and FPOWA. The framework enables the SMEs to choose the right type of social commerce considering TOE factors that help them build a stronger relationship with current and potential customers. The approach's applicability is demonstrated using a case study of three SMEs seeking to adopt a social commerce type. The analysis results indicate the proposed approach's effectiveness in handling uncertain, complex nonlinear decisions in social commerce adoption.

## Introduction

Small and Medium-sized Enterprises (SMEs) refer to the business having a limited number of working personals and resources. The definition of SME varies from one country to another based on the state’s level of development. For example, small-sized enterprises in Turkey and the European Union have less than 50 and middle-sized businesses with less than 250 (Bassi and Dias 2020). However, small businesses have less than 20 employees in Australia, and medium enterprises have employees between 20 and 199 (Bakhtiari et al. 2020). SMEs constitute a remarkable role in encouraging economic competitiveness, employer contributions and entrepreneurial improvements. According to the World Bank, SMEs represent 90% of business and 50% of employment worldwide (Bank 2021). For both developing and developed countries, SMEs are the building blocks of a state’s economy and the primary drivers for a country’s GDP growth and socio-cultural development. In Australia, SMEs contribute 42.4% of the Australian GDP, nearly 98% of Australian businesses and employ around 44% of the Australian workforce (Business and Ombudsman 2020). Despite these promising contributing figures, SMEs face many challenges that cause many companies to cease before operating. One of SMEs' significant challenges is the lack of digital and digital media to approach new consumers (Kabanda and Brown 2017).

E-commerce provides an opportunity for global reach, a low barrier to trade, better cost-saving, accessibility and an ideal kick-start for small businesses in challenging situations like Covid-19 (Gao et al. 2022). The effectiveness of business productivity gets double when E-commerce features are combined with social media named Social Commerce (SC) (Lin et al. 2017). The SC is rapidly evolving, radically boosting online consumers' shopping experience and providing a seamless e-commerce experience while using social channels. Social commerce offers many business benefits that range from increasing consumer’s engagement, easy electronic payment, convenience, and website traffic. The critical feature of social commerce is its feature of shopping with social experience. A buyer can interact and exchange their experiences with their friends' network and shop instantly regardless of physical location. According to one of the latest surveys by Statista (Tugba 2020), the social commerce sales in the USA were US$22 billion that reached US$ 29.3 billion and are expected to reach US$84.2 billion in 2024. In another survey (Duong 2020), in 2020, the active social media users were over 3.6 billion worldwide, reaching 4.41 billion in 2025. Similarly, in many countries like Australia, 63% of SMEs have social media accounts, with 91% handling their social media profile (Business and Ombudsman 2020).

Social commerce is divided into two categories—onsite social commerce and offsite social commerce. Onsite social commerce includes all social functionality on a company’s website, including consumers' recommendations, visual photos, or social proofs (Zhang and Benyoucef 2016). Some examples of onsite social commerce are *j.crew.com,* which places consumer’s feedback front and centre on each product page. Sephora.com, an online fragrance store, offers a “Fragrance IQ” quiz to get insight into consumers' personalised choices. The quiz covers small brief questions to assist a consumer in determining the ideal perfume. Unlike onsite SC, offsite SC includes social activities outside of the company’s website. Businesses usually use—Facebook, Instagram, Twitter, Pinterest and many other social networking websites.

Recent COVID-19 lockdown has transformed the use of technology for SMEs. The closure of physical premises makes a compulsive adoption of digital technology and a new way to interact and stay connected with consumers. One of the best ways to deal with the situation is the adoption of SC. When SMEs have decided to strategically integrate SC into their business, the next question is adopting onsite or offsite social commerce. The adoption strategy depends on several factors such as—advertisement, brand exposure, better interaction, an avenue for contact and effective tool. In the latest survey by Yellow Social Media, 22% of Australian SMEs are concerned with offsite SC adoption because of negative reviews or ratings online (Olanrewaju et al. 2020). However, many other small and medium businesses prefer offsite SC due to wider social coverage, better visibility and cost savings. Existing literature evaluate the adoption of social commerce from a different perspective. Some of the areas are – trusted relationship (Algharabat and Rana 2020; Bugshan and Attar 2020) between consumer and provider, consumer collaboration (Osatuyi et al. 2020), better purchase decision (Lăzăroiu et al. 2020) and finding right product and service providers (Alkalbani and Hussain 2021; Alkalbani et al. 2019; Lin et al. 2017). However, there is very limited literature on adopting onsite, offsite or hybrid SC adoption strategies for SMEs.

This study evaluates the problem in terms of the multi-criteria group decision making (MCGDM) process. The MCGDM has widely used a decision-making strategy that combines multi-criteria decision making (MCDM) and group decision making approaches (Hussain and Merigó, 2022; Petchimuthu et al. 2020). Naeem et al. (2019) proposed a hybrid MCGDM method by combining techniques for order of preference by similarity to ideal solution (TOPSIS) and VIekriterijumsko KOmpromisno Rangiranje (VIKOR) method for stock exchange recommendations. Hussain et al. (2021a, b; 2022a, b) introduced an OWA layer in the prediction method to handle complex nonlinear prediction (Hussain et al. 2022a). Yuan et al. (2021) proposed a hybrid method by combining DEcision MAking Trial And Evaluation Laboratory (DEMATEL) and COmplex PRoportional Assessment (COPRAS) method to handle the decision-making process in logistic providers selection. Musso and Francioni (Musso and Francioni 2012) analysed various aspects of SME’s decision making process by considering various organisations in Italy. The study revealed that there is a positive relationship between the educational background of the decision-maker with the market selection and entry mode selection. Wang et al. (2021) proposed the MCGDM method—MULTIMOORA and adopted a probabilistic linguistic term set to evaluate the trust of the decision-makers. Zhang et al. (2021a, b) proposed multi-granular unbalanced linguistic terms to assist the decision-maker in reaching consensus in group decision-making problems. Zhang et al. (2021a, b) proposed a two-sided matching problem with a hesitant fuzzy linguistic term set. The authors used a multi-granular hesitant fuzzy linguistic term set for the decision making process. Kacprzyk et al. (2019) proposed a human-centric aggregation by using the OWA operator to aggregate numerical values in social choice results. Alfaro-García et al. (2021) proposed a group decision-making model using ordered weighted logarithmic aggregation operators. The approach analysed the strategic decision-making process in a multi-person analysis. In another approach, Wen and Liao (2021) analysed attitudinal characteristics of the decision-maker by considering the probabilistic linguistic term set. Garg et al. (2021) proposed a fuzzy power aggregation operator for the T-spherical fuzzy sets for the multi-attribute decision-making process. Akram et al. (2021) proposed a hesitant Pythagorean fuzzy set and combined it with the ELECTRE-II method to define the outranking relations in the MCGDM problem. Different machine learning method such as Połap et al. (2021) and Hussain et al., (2018a, b) are used to assist the stakeholder in decision making process. Although discussed approaches assist the decision-making process, the approaches are unable to unify all possible attitudinal behaviour of the decision maker in one single formulation.

We propose a novel hybrid MCGDM method to address the discussed problem. In this approach, we combine Fuzzy Analytical Hierarchical Process (FAHP) with the OWA operator, enabling the decision-maker to decide uncertainty. We employ the Technology–Organisation–Environment (TOE) framework (Tornatzky and Fleischer 1990) and evaluate their attributes using the OWA operator for the best alternative. The decision-making process is from the context of benefits where the decision-maker wants to maximise the benefits. Different service providers have different attitudinal behaviour to take a possible risk of technology adoption. Recommended action varies for the service provider who has a risk-taking behaviour to the risk-averse behaviour. Therefore, one type of recommended action is not practicable for all situations. Unlike previous approaches, the proposed approach can unify all possible attitudinal behaviour of the decision-maker in a single formulation to best describe the decision-making process. In the existing literature, no method could fit all attitudinal behaviour of the decision-maker to choose onsite/offsite or hybrid social commerce adoption. The study aims to analyse the SME manager’s decision-making process, where the available data is imprecise that can be evaluated using fuzzy numbers. The approach was studied using a multi-person fuzzy OWA aggregation operator. Several particular cases—Fuzzy Minumum (FMin), Fuzzy Maximum (FMax), Laplace Criteria, Hurwicz criteria, Fuzzy Weighted Average (FWA), Fuzzy Ordered Weighted Average (FOWA), Fuzzy Probabilistic Ordered Weighted Average (POWA) evaluated to demonstrate different attitudinal characteristics of the decision-maker. The system's output is onsite/offsite/hybrid SC adoption recommendation under the uncertain, complex framework. The distinctive feature of this paper is as follows:

- The paper proposes a novel framework to assist SMEs on onsite/offsite/hybrid social commerce adoption strategies.
- This is the first work that enables SME managers to take an optimal decision for SC adoption in a complex nonlinear structure where the available information is imprecise and fuzzy.
- Many existing studies enable SMEs for social commerce adoption. However, to the best of the author knowledge, this is the first work that uses the TOE framework in a multi-criteria group decision making using OWA operator for onsite/offsite/hybrid social commerce adoption.
- Unlike existing approaches, the proposed framework helps the decision making under uncertainty and evaluate various attitudinal characteristics of the decision-makers such as – optimistic, pessimistic or neutral.
- The approach's applicability and effectiveness are demonstrated using a case study where SMEs assess factors for optimal adoption of social commerce.

The rest of the paper is organised as follows. Section 2 discusses some background and related literature on social-commerce, TOE, fuzzy linguistic approach for criteria weights and aggregation operator. Section 3 presents the methodology followed by a case study in Sect. 4. Section 5 presents the proposed approach's evaluation and implementation, and Sect. 6 concludes the paper with future research directions.

## Theoretical background/literature review

This section discusses some background and related studies on social commerce, TOE framework, MCDM, OWA aggregation operator and families.

### Social commerce

Social media marketing is part of a broader advertising communication strategy for an effective medium to exchange—user experience, more comprehensive brand advertisement and effective communication. Social commerce is the next generation of e-commerce that has a strong influence on online business. The critical factor in SC is consumers' purchasing intention based on social opinion with social proofs, unlike a traditional advertisement, where the company only presents product features. This emerging era of e-commerce has grasped the attention of many researchers. Bugshan and Attar (2020) highlights the trust in sharing SC information sharing and its impact on privacy and buying decision. Authors draw a conceptual model based on five hypotheses, collected and analysed data using PLS-SEM techniques. Results reveal that SC information sharing boosts up trust in sharing commerce decreases perceived privacy risk and improves intentions to buy decision-making processes. In a similar approach, Algharabat and Rana (2020) analyse SC's impact on online community engagement and individual member’s trust. Authors found that SC constructs positive individuals’ trust in the community and constructive community engagement. In another approach, Al-Tit, Omri and Hadj (2020) investigated multiple factors which drives up the SC intention of online communities. The authors considered various factors such as—social support, trust, social commerce intention and constructs to draw a relationship. The study found that trust conciliates social support with social commerce intentions. Moreover, the SC constructs are directly related to SC intentions and emotion information support that boost up social commerce intentions among consumers. Commonly many customers are reluctant to shift to social commerce to gain a new shopping experience. To investigate the issue, Changchit, Cutshall and Pham (2020) analysed consumers’ social commerce intention from demographic and personal characteristics to understand the reasons for reluctance. They found that demographic and personality correlate in the adoption of social commerce. Younger users under the age of 36 prefer using social commerce compare to other age groups. Osatuyi et al. (2020) proposed a model to define quadratic relationship mong antecedents of different social commerce constructs. Authors considered different constructs such as perceived usefulness, satisfaction, confirmation and continuance intention to study non-linear and inverted U-relationships among the determinants. The study found that continuance intention has a non-linear link to perceived usefulness through satisfaction. With respect to gender classification, perceived usefulness interacts positively with satisfaction for females. For males, the association is inverted U-relationship. Alamgir and Minho (Hossain & Kim 2020) surveyed 549 participants to analyse the user experience of social networking websites. The study found that a service quality of social networking websites has a positive impact on consumer’s satisfaction and increased social capital. This will lead the acceptance of social commerce. Moreover, the study found that perceived trust acts as a mediator between usage intention and social commerce intention.

### Technology-Organisation-environment (TOE) framework

TOE framework was introduced by Tornatzky and Fleischer (1990). It describes many factors which influence the adoption of new technology and its likelihood. This paper considered TOE attributes to predict the possible adoption of onsite/offsite/hybrid SC for SMEs. The adoption strategy is subjective to technological context, organisational context and environmental context.

The TOE is a widely used framework for the adoption strategy of various technologies across different industries. Sikandar et al. (2020) evaluated social media adoption and its impact on SMEs performance using the TOE framework. The study found that social media positively impact SMEs performance. Moreover, TOE factors such as interactivity, relative advantage, and top management support directly influence SMEs' social media adoption. Cruz-Jesus et al. (2019) applied TOE framework to assess the antecedents of CRM adoption. The study found that factors—technology competence, data quality, top management support and CRM evaluation have a positive impact, while competitive pressure has a negative effect on CRM adoption. Tiago et al. (2019) applied the TOE framework and institutional theory to investigate the environment factor in adopting onsite software as a service (SaaS). The results found that technology and environment factors have the moderator influence between an organisation and SaaS adoption that directly impacts onsite adoption procedure. Abed (2020) conducted an empirical study to analyse SC adoption by SMEs using TOE framework. The study found that trading partner pressure, top management support, and perceived usefulness significantly impact SC adoption. In another study, Lorente-Martínez et al. (2020) analysed the adoption of in-store technology adoption by SMEs using TOE and TAM framework. The study found that top-management support is the strong predictor of adopting technology in the organisation.

A brief explanation and definition of each factor are presented in the below section.

#### Technology

The technology factor in TOE describes the internal and external technologies appropriate to the organisation, which might increase the organisation's productivity. Advances in technology transform the way of doing business and have a crucial position in the business process. Specifically, it is challenging for SMEs with limited budgets and resources to decide the right type of technology adoption. Technology has the potency to positively and negatively impact SMEs, depending on their adoption strategy (Tiago et al. 2019). Most commonly used (Boumediene et al. 2009; Sohaib et al. 2019) technology factors are—relative advantage, complexity, compatibility, security/privacy, reliability and scalability. Table 1 define each of the technology factors briefly.

**Table 1:** Technology factors of TOE framework with reference of onsite/offsite SC adoption

Factor	Definition
Relative advantage	Roger (2010) defined relative advantage as the degree to which a consumer finds a new product or service better than its substitute. SC add value to the business with its broader scope, effective interaction, social communication and recommendation (Huang and Benyoucef 2013)
Complexity	Complexity is defined as “the degree to which an innovation is perceived as relatively difficult to understand and use” (Rogers 2010). While adopting onsite or offsite SC, a different group of users may experience complexity, ultimately creating uncertainty for successful adoption
Compatibility	Compatibility is defined as “the degree to which an innovation is perceived as consistent with the existing values, past experience and needs of potential adopters” (Rogers 2010). This is an essential factor in SC adoption. It defines how SC makes significant work practice changes, which are compatible with their values and beliefs (Boumediene et al. 2009)
Security / Privacy	Security and privacy define the safeguard of consumer’s data and their identity from unauthorised access and use. Privacy assurance is one of the key features in SC websites that help build a trusted relationship between stakeholders. For adopting an onsite or offsite SC, the decision-makers need to assess which option gives better security and privacy to win a consumer’s trust, consequently increasing product purchase likelihood (Wang and Herrando 2019)
Reliability	Reliability defines the accuracy degree of consumer’s feedback and their sentiment regarding offered services and products (Raza et al. 2021a, b)
Scalability	Scalability defines the ability to compute, process, store, communicate and transfer multiple types of data across the network when the number of consumers or offered services increases (Hussain et al. 2015)

#### Organisation

The organisation factor in TOE defines the readiness of the organisation and top-management support to adopt a technology (Rogers 2010). The organisation factors used in the study are—organisational readiness, firm size and top management support (Boumediene et al. 2009; Sohaib et al. 2019). Table 2 define each of the organisation factors briefly.

**Table 2:** Organisation factors of TOE framework regarding onsite/offsite SC adoption

Factor	Definition
Organisational readiness	It defines the availability of necessary IT infrastructure and human resources with the required skills to perform the task and financial resources for adoption (Sikandar Ali et al. 2020)
Firm size	Firm size is an essential factor in technology adoption (Cruz-Jesus et al. 2019). The choice decision of onsite/offsite significantly varies among small, medium and large enterprises. Many large enterprises are reluctant to adopt offsite SC, because poor performance reviews have attributed to the lack of buying intent (Duong 2020) of potential buyers
Top management support	The support of the top management creates a productive environment for the adoption of new technology. The top management is more concerned with the most productive, cost-efficient solutions that increase the revenue with existing or limited resources (Jeyaraj et al. 2006)

#### Environment

The environment factor highlights different external environmental pressure that directly or indirectly impacts the adoption of technology. The adoption strategy highly depends on the availability of technology service providers, government legislation regarding technology use, and existing rivalry (Huang and Benyoucef 2013). This paper has considered three environmental factors – competitive pressure, trading partner pressure, and government regulations, as presented in Table 3.

**Table 3:** Environment factors of TOE framework regarding onsite/offsite SC adoption

Factor	Definition
Competitive pressure	It defines the degree of competition an organisation faces in the adopter’s industry. The competitive pressure is directly proportional to the adoption of the new technology (Huang and Benyoucef 2013)
Trading partner pressure	The readiness of the business partner and internet technology supplier significantly impact the adoption of the SC. The higher powerful suppliers with greater expertise have a greater influence of the adoption of technology (Lorente-Martínez et al. 2020; Rogers 2010)
Government regulations	SC adoption highly depends on government legislation regarding the use of technology. For example, Australian authorities have a very close eye on the video-sharing social website TikTok and may ban in future due to security interests (Fouskas et al. 2021)

### Linguistic approach for criteria weights and aggregation operators

In this section, the paper briefly discusses the Fuzzy Numbers (FN), the Fuzzy Weighted Average (FWA), Fuzzy Ordered Weighted Average (FOWA)linguistic approaches, Fuzzy Analytical Hierarchical Process (FAHP).

#### The linguistic approach

**Fig. 1:**
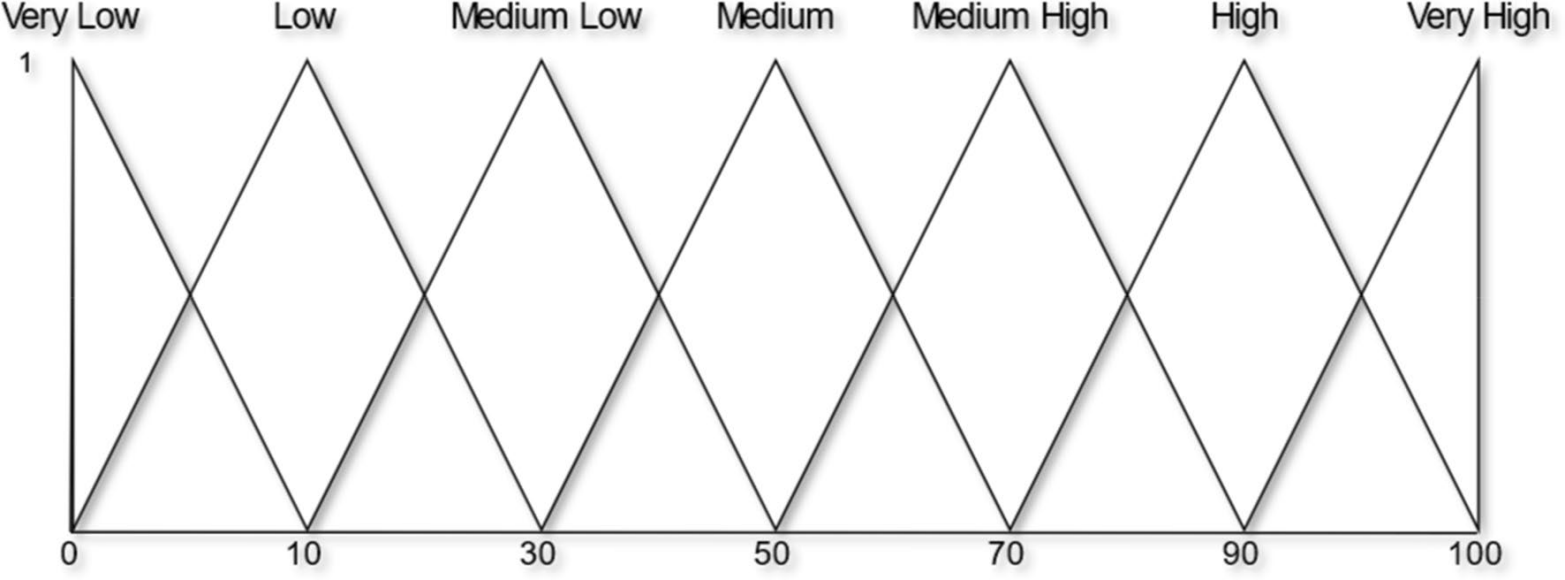
Linguistic variables and membership function

In a real-life situation, the quantitative assessment is not possible due to vague or imprecise knowledge. In those situations, the decision-makers often use qualitative assessment by using linguistic variables. This paper considers seven linguistic terms that best present decision-makers judgment criteria (Sohaib et al. 2019). The set of seven linguistic terms $S= \left\{VL,L,ML,M,MH,H,VH\right\}$ with their TFN is presented in Table 4 and Fig. 1.

**Table 4:** Linguistic term and fuzzy numbers

Linguistic term	Fuzzy number
Very low (VL)	(0, 0, 10)
Low (L)	(0, 10, 30)
Medium low (ML)	(10, 30, 50)
Medium (M)	(30, 50, 70)
Medium high (MH)	(50, 70, 90)
High (H)	(70, 90, 100)
Very high (VH)	(90, 100, 100)

#### Fuzzy numbers (FNs)

The FN was introduced by Zadeh (1975) and has been studied in a wide range of applications (Hussain et al. 2018a, b; Hussain et al. 2020; Sohaib et al. 2019). The FN is defined as:

##### Definition 1

*A fuzzy number* ${F}^{\sim }$ *on* ℝ *is a fuzzy subset of the real line where *${\mu }_{{F}^{\sim }}\left(\lambda {a}_{1}+ \left(1- \lambda \right){a}_{2}\right) \ge \mathrm{min}\left({\mu }_{{F}^{\sim }}\left({a}_{1}\right), {\mu }_{{F}^{\sim }}\left({a}_{2}\right)\right)$* for *$\forall {a}_{1}, {a}_{2} \in {\mathbb{R}} \mathrm{and }\lambda \in \left[\mathrm{0,1}\right]$*.*There is a broad range of FNs in literature (Hussain et al. 2016; Merigo et al. 2014), such as triangular, trapezoidal FN, generalised FN, interval-valued FN and other complex structures. In this paper, we use fuzzy triangular fuzzy numbers (TFN) to capture the vagueness of the linguistic assessments (Gani and Assarudeen 2012). For example, a TFN Ã = (${a}_{1},{a}_{2},{a}_{3}$) of a universe of discourse ℝ can be characterised by triangular membership function that satisfies following conditions:

- a_1,_ a_2_ is an increasing function
- a_2,_ a_3_ is a decreasing function
- a_1 ≤_ a_2 ≤_ a_3_

The α-cut representation of Ã $\left(\underline{a}, \overline{a }\right)$ $\forall \alpha \in \left[\mathrm{0,1}\right]$ parameterised by (${a}_{1},{a}_{2},{a}_{3}$) such that

1 $$ \begin{aligned} &\underline{a} \left(\alpha \right)= {a}_{1}+ \alpha \left({a}_{2} - {a}_{1}\right) \\ &  \overline{a} \left(\alpha \right)= {a}_{3} - \alpha \left({a}_{3} - {a}_{2}\right) \end{aligned}$$

The TFN is represented as follows:

2 $${\mu }_{\mathrm{\tilde{A} }}\left(x\right)= \left\{\begin{array}{c}0 for x< {a}_{1}\\ \frac{x-{a}_{1}}{{a}_{2}- {a}_{1}} for {a}_{1}\le x \le {a}_{2}\\ \begin{array}{c}\frac{{a}_{3}-x}{{a}_{3}-{a}_{2}} for {a}_{2}\le x \le {a}_{3}\\ 0 for x< {a}_{1}\end{array}\end{array}\right. $$

#### Fuzzy weighted average (FWA)

The FWA is an extension of the weighted average that deals with uncertain information. It can be defined as follows (Casanovas and Merigo 2012):

##### Definition 2

*Let *$\xi$* be a set of fuzzy numbers. A FWA operator of dimension m is a mapping function FWA: *${\upxi }^{k}\rightarrow \xi$*, which has an affiliated group of weighting vector W of dimension m, such that *$\sum_{j=1}^{m}{w}_{j}=1$* and *${w}_{j}\in \left[\mathrm{0,1}\right]$*,* such that:

3 $$FWA ( {\check{x}}_{1}, {\check{x}}_{2}, {\check{x}}_{3}, \dots \dots ., {\check{x}}_{m})=\sum_{j=1}^{m}{w}_{j}{\check{x}}_{j} $$

where $\check{x}_{i}$ are the FNs.

#### Fuzzy ordered weighted average (FOWA)

The FOWA is an extension of OWA operator (Yager 1988) that considers uncertain information and aggregate multiple inputs residing between two extremes. The FOWA provides a parameterised family of aggregation operators that include fuzzy minimum (FMin), fuzzy maximum (FMax) and fuzzy average (FA) and others. It can be defined as:

##### Definition 3

*Let *$\Psi$* be a set of fuzzy numbers *$( {\check{a}}_{1}, {\check{a}}_{2}, {\check{a}}_{3}, \dots \dots ., {\check{a}}_{n})$*. A FOWA operator of dimension n is a mapping FOWA: *${\Psi }^{n}\rightarrow\Psi$*, which has an affiliated group of weighting vector W of dimension n, such that *$\sum_{j=1}^{m}{w}_{j}=1$* and *${w}_{j}\in \left[\mathrm{0,1}\right]$*,* such that:

4 $$FOWA ( {\check{a}}_{1}, {\check{a}}_{2}, {\check{a}}_{3}, \dots \dots ., {\check{a}}_{n})=\sum_{j=1}^{n}{w}_{j}{\check{b}}_{j} $$

where $( {\check{b}}_{1}, {\check{b}}_{2}, {\check{b}}_{3}, \dots \dots ., {\check{b}}_{n})$ are the reordered set of $( {\check{a}}_{1}, {\check{a}}_{2}, {\check{a}}_{3}, \dots \dots ., {\check{a}}_{n})$ FNs from largest to smallest.

#### Fuzzy probabilistic ordered weighted average (FPOWA)

The probabilistic OWA (POWA) operator (Merigó, 2010) is one of the OWA operator families that combines the decision-makers probabilistic and attitudinal characteristics under one formulation. The POWA operator is defined as follows:

##### Definition 4

*Let* $\stackrel{\sim }{\Pi }$ *be a set of fuzzy numbers*$( {\check{a}}_{1}, {\check{a}}_{2}, {\check{a}}_{3}, \dots \dots ., {\check{a}}_{n})$*.* A *FPOWA operator of dimension n is a mapping FPOWA:*${\stackrel{\sim }{\Pi }}^{n} \rightarrow \stackrel{\sim }{\Pi }$* that has an associated group of weighting vectors W of dimension n such that *$\sum_{i=1}^{n}{w}_{i}=1$* and *${w}_{i}\in \left[\mathrm{0,1}\right]$* and a probabilistic vector P such that *$\sum_{k=1}^{n}{p}_{k}=1$* and *${p}_{k}\in \left[\mathrm{0,1}\right]$*, such that:*

5 $$FPOWA \left( {\check{a}}_{1}, {\check{a}}_{2}, {\check{a}}_{3}, \dots \dots ., {\check{a}}_{n}\right)=\gamma \sum_{i=1}^{n}{w}_{i}{\check{b}}_{j}+ \left(1-\gamma \right)\sum_{k=1}^{n}{p}_{k}{\check{a}}_{k} $$

where $( {\check{b}}_{1}, {\check{b}}_{2}, {\check{b}}_{3}, \dots \dots ., {\check{b}}_{n})$ are the reordered set of $( {\check{a}}_{1}, {\check{a}}_{2}, {\check{a}}_{3}, \dots \dots ., {\check{a}}_{n})$ FNs from largest to smallest and $\gamma \in [0, 1]$.

#### Fuzzy analytical hierarchical process (FAHP)

Buckley (1985) introduced the geometric mean method to extend the AHP that can deal with the linguistic variables. To calculate the fuzzy relative importance among criteria, the FAHP construct a pairwise comparison matrix ${\check{\uppsi}}= \left[{\check{x}}_{i,j}\right]$ as follows:

6 $${\check{\uppsi}}=\left[\begin{array}{c}\left(\mathrm{1,1},1\right)\\ \begin{array}{c}{\check{x}}_{\mathrm{2,1}}\\ \begin{array}{c}\vdots \\ {\check{x}}_{n,1}\end{array}\end{array}\end{array} \begin{array}{c}{\check{x}}_{\mathrm{1,2}}\\ \begin{array}{c}\left(\mathrm{1,1},1\right)\\ \begin{array}{c}\vdots \\ {\check{x}}_{n,2}\end{array}\end{array}\end{array} \begin{array}{c}\begin{array}{c}\cdots \\ \cdots \end{array}\\ \ddots \\ \cdots \end{array} \begin{array}{c}{\check{x}}_{1,n}\\ \begin{array}{c}{\check{x}}_{2,n}\\ \begin{array}{c}\vdots \\ \left(\mathrm{1,1},1\right)\end{array}\end{array}\end{array} \right] $$

where$${\check{x}}_{i,j}\times {\check{x}}_{\mathrm{1,2}} \approx 1\mathrm{ and }{\check{x}}_{\mathrm{1,2}} \cong \frac{{w}_{i}}{{w}_{j}} , i,j=\mathrm{1,2},\dots ., n$$.

The geometric mean ${\check{\varphi}}$ for individual criteria, *i* is computed as:

7 $${\check{\varphi}}= {\left({\check{x}}_{i,1}\times {\check{x}}_{i,2}\times \cdots \times {\check{x}}_{i,3}\right)}^{1/n}$$

The approach then calculates the fuzzy weight for each criterion *i* using the equation below:

8 $${\check{w}}={{\check{\varphi}}}_{i} {\times \left({{\check{\varphi}}}_{1}+ {{\check{\varphi}}}_{2}+ \cdots + {{\check{\varphi}}}_{3}\right)}^{-1} $$

where $${{\check{\varphi}}}_{j}= \left({l}_{j}, {m}_{j}, {u}_{j}\right)$$ and$${\left({{\check{\varphi}}}_{j}\right)}^{-1}= \left(\frac{1}{{u}_{j}}, \frac{1}{{m}_{j}} ,\frac{1}{{l}_{j}}\right)$$.

To defuzzify the fuzzy weights ${{\check{w}}}_{j}= \left({l}_{j}, {m}_{j}, {u}_{j}\right)$ the approach calculates the centre of the area (CoA) using the following equation:

9 $${{\check{w}}}_{j}= \frac{\left({l}_{j}, {m}_{j}, {u}_{j}\right)}{3} $$

## Methodology

**Fig. 2:**
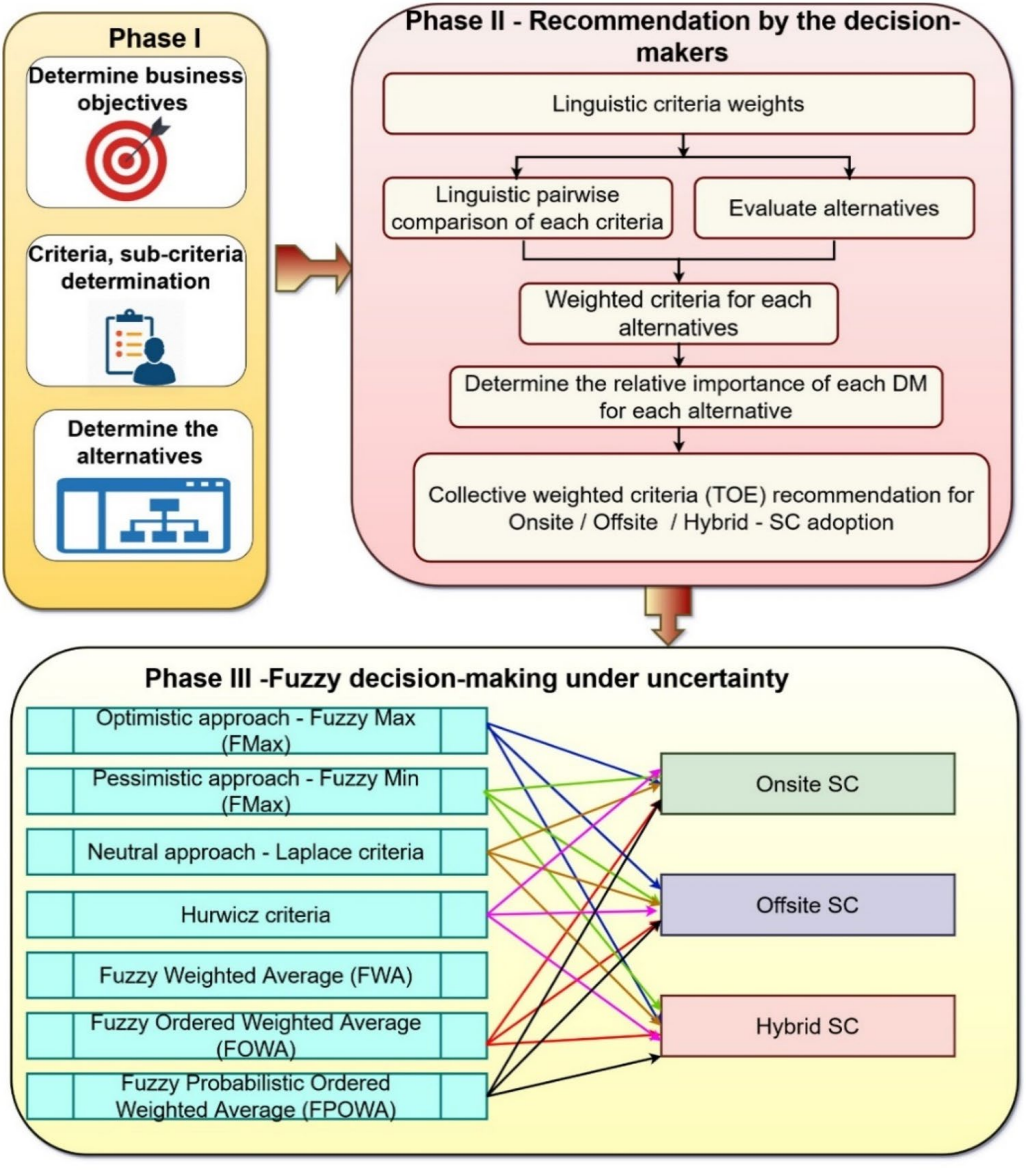
Proposed linguistic MCGDM Framework

This section presents the proposed fuzzy hybrid FAHP and FOWA approaches using FNs on the MCGDM process. The proposed system is divided into the following three phases, as presented in Fig. 2. The working of each phase is presented below:

### Phase I: Defining the business goal and key attributes

**Fig. 3:**
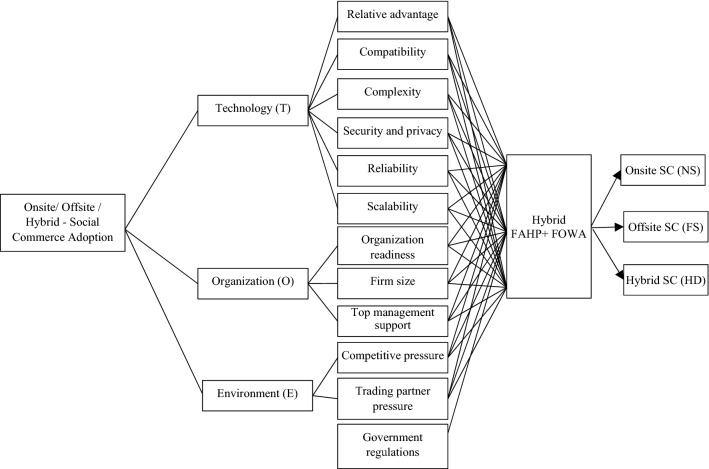
Hierarchical structure of the decision problem

The primary objective of the executives is to adopt the best social commerce alternatives for SMEs. The decision-makers also want to know the best alternative under complex, uncertain different attitudinal individualities. For an optimal decision-making process, it is vital to identify and assess the proper set of attributes and their sub-attributes that portray an enterprise's holistic picture. In this study, we have adopted the TOE framework used in various literature (Lorente-Martínez et al. 2020; Sikandar Ali et al. 2020; Tiago et al. 2019) for a technology adoptions. The TOE framework has three criteria—technology, organisation and environment that further splits into twelve sub-criteria. The system evaluates the impact of TOE criteria on SMEs SC adoption strategy. The system applies hybrid FAHP to ascertain the relative weights of each criterion and apply linguistic OWA approaches to recommend onsite/offsite/hybrid SC. Moreover, to present the decision-makers' different attitudinal characteristics, the system uses several proposed approaches. The hierarchical structure of the proposed approach with the breakdown structure of business goal, attributes, sub-attributes, approach and alternatives is presented in Fig. 3.

### Phase II: Recommendation Inputs by the decision-makers (DMs)

After defining the business goal, identifying key attributes, sub-attributes, and all alternatives, the system seeks DMs recommendation for adopting adoption. In this phase, the system grouped decision-makers into multiple categories from various departments that understand SC and its impact on their respective department. The working of these phases is summarised as follows:

*Step 1:* Let $DM$ be a set of decision-makers $DM= \left\{{DM}_{1},{DM}_{2},\dots ..,{DM}_{n}\right\}$ that evaluate the importance of each criterion $CR= \left\{{Cr}_{1},{Cr}_{2},{Cr}_{3},\dots ..{Cr}_{n}\right\}$ to select the best alternative $A= \left\{{alt}_{1}, {alt}_{2},\dots \dots ., {alt}_{n}\right\}$.

*Step 2:* Each element of criteria $CR$ have a different degree of importance for DMs. To determine the relative importance of $CR$, the system uses FAHP. It enables to obtain the pairwise comparison matrix and determine criteria weights ${\widehat{CR}}_{w}= \left\{{\widehat{Cr}}_{w1},{\widehat{Cr}}_{w2},{\widehat{Cr}}_{w3},\dots ..{\widehat{Cr}}_{wn}\right\}$. The system uses the relative importance range for criteria weights, as presented in Table 5.

**Table 5:** Relative importance for criteria weights in linguistic term

Relative importance	Crisp value	Triangular fuzzy value	Triangular fuzzy reciprocal scale
Equal	1	(1,1,1)	(1,1,1)
Weak or slight	2	(1,2,3)	(1/3,1/2,1)
Moderate	3	(2,3,4)	(1/4,1/3,1/2)
Moderate plus	4	(3,4,5)	(1/5,1/4,1/3)
Strong	5	(4,5,6)	(1/6,1/5,1/4)
Strong plus	6	(5,6,7)	(1/7,1/6,1/5)
Very strong	7	(6,7,8)	(1/8,1/7,1/6)
Very, very strong	8	(7,8,9)	(1/9,1/8,1/7)
Extremely strong	9	(9,9,9)	(1/9,1/9,1/9)

*Step 3:* Each decision-maker $DM$ provide its recommendations $\tilde{RC }=\left\{{\tilde{R }C}_{1}, {\tilde{R }C}_{2}, {\tilde{R }C}_{3}, \dots ., {\tilde{R }C}_{n}\right\}$ for each criterion and sub-criteria towards the selection of alternative $A$. The linguistic range of importance is presented in Table 4.

*Step 4:* The relative criteria weights ${\widehat{CR}}_{w}$ regarding each recommendation $\tilde{RC }$ is applied to get weighted criteria recommendation ${\check{WCR}}$ for each alternative $A$, as presented in the below equation:

10 $$\forall CR \ni {{\check{WCR}}}_{i}=\left[{\widehat{CR}}_{wi} \times {\tilde{RC }}_{i}\right], i\in \left[1,n\right] $$

where $n$ is the total number of criteria and sub-criteria.

*Step 5:* Depending on previous experience, influence in an organisation, and other factors, each DM's decision has different importance in the decision-making process. Let ${DM}_{w}= \left\{{\overline{DM} }_{w1}, {\overline{DM} }_{w2}, \dots ., {\overline{DM} }_{wn}\right\}$ is a set of weight vector for each DM. To include the relative importance of DM in a decision-making process, the system applies the relative importance of decision-makers ${DM}_{w}$ on weighted criteria recommendation $WCR$. As a result, the system gets weighted criteria decision-maker recommendation $\overline{WCDR }$ for each alternative $A$, as presented in the below equation:

11 $$\forall {\check{WCR}} \ni \overline{WCDR }=\left[{{\check{WCR}}}_{i} \times {\overline{DM} }_{wi}\right], i\in \left[1,n\right] $$

where $n$ is the total number of criteria and sub-criteria for all alternatives.

*Step 6:* The system uses the weighted average to determine the collective aggregated information ${\tilde{CAI }}_{TOE}$ in relation to Technology ($T$), Organisation ($O$) and Environment ($E$) factors provided by decision-makers $DM$ for each alternative $A$.

12 $${\tilde{CAI }}_{TOE}=\sum_{i=1}^{n}{\overline{WCDR} }_{i}\left(T\right) , \sum_{i=1}^{n}{\overline{WCDR} }_{i}\left(O\right), \sum_{i=1}^{n}{\overline{WCDR} }_{i}\left(E\right) $$

where $n$ is the total number of criteria and sub-criteria for all alternatives.

### Phase III: Fuzzy decision-making under uncertainty

This section presents the fuzzy multi-criteria group decision-making (FMCGDM) under uncertainty, and there is no probabilistic information to assess it. The FOWA aggregation operator is used to handle imprecise uncertain information in the decision-making process. Let $W= \left({w}_{1},{w}_{2}, {w}_{3}, \dots ., {w}_{n}\right)$ be the OWA weight vector such that $\sum_{i=1}^{n}{w}_{i}=1, {w}_{i}\in \left[\mathrm{0,1}\right]$. We analyse the decision making process from the context of benefits, i.e. to maximise the benefits. Depending on the decision-makers' attitudinal characteristics, the following methods are applied that best present the DMs' propensity.

- **Fuzzy optimistic approach (FMax):** In this case, the decision-maker is very optimistic. Therefore we select the highest result for each alternative obtained, as presented in the below equation.
  13 $${w}_{1}=1, {w}_{j}=0\mathrm{ for }j \ne 1 \Rightarrow Max \left({a}_{i}\right) $$
- **Fuzzy pessimistic approach (FMin):** In this case, the decision-maker is very pessimistic against the future. The approach makes safety decisions, that can guarantee the minimum results. Here we select the lowest result for each alternative, as presented in the below equation.
  14 $${w}_{n}=1, {w}_{j}=0\mathrm{ for }j \ne n \Rightarrow Min \left({a}_{i}\right) $$
- **Laplace criteria:** In this case, we assume a neutral approach in which all state of nature is equally important. Therefore, we calculate the arithmetic mean of available criteria for each alternative, as presented in the below equation.
  15 $${w}_{i}= \frac{1}{n} for all i \Rightarrow \left(\frac{1}{n}\right)\times \left(\sum {a}_{j}\right) $$
- **Hurwicz criteria:** In this case, we consider that the decision-maker has a certain degree of optimism $\alpha$ and a certain degree of pessimism. The decision making under this approach is presented as follows:
  16 $${w}_{1}=\alpha , {w}_{n}=\left(1- \alpha \right) and {w}_{j}=0\mathrm{ for }j \ne 1,n \Rightarrow \alpha \times Max \left\{{a}_{i}\right\} + \left(1- \alpha \right) \times Min \left\{{a}_{i}\right\} $$
- **Fuzzy Weighted Average (FWA):** In this case, the decision-maker evaluates each alternative based on a fixed number of states with known probabilities. In this case, the DMs define individual weights for each criterion and alternatives being assessed based on that, as presented in Eq. 3.
- **Fuzzy Ordered Weighted Average (FOWA):** In this approach, we use the OWA weights and determined each alternative based on multiplying weights with the reordered inputs from largest to smallest, as presented in Eq. 4.
- **Fuzzy Probabilistic Ordered Weighted Average (FPOWA):** This approach blends the decision maker’s probabilistic and attitudinal characteristics in a single formulation, as presented in Eq. 5.

The algorithmic representation of the decision-making process is illustrated in Algorithm 1.
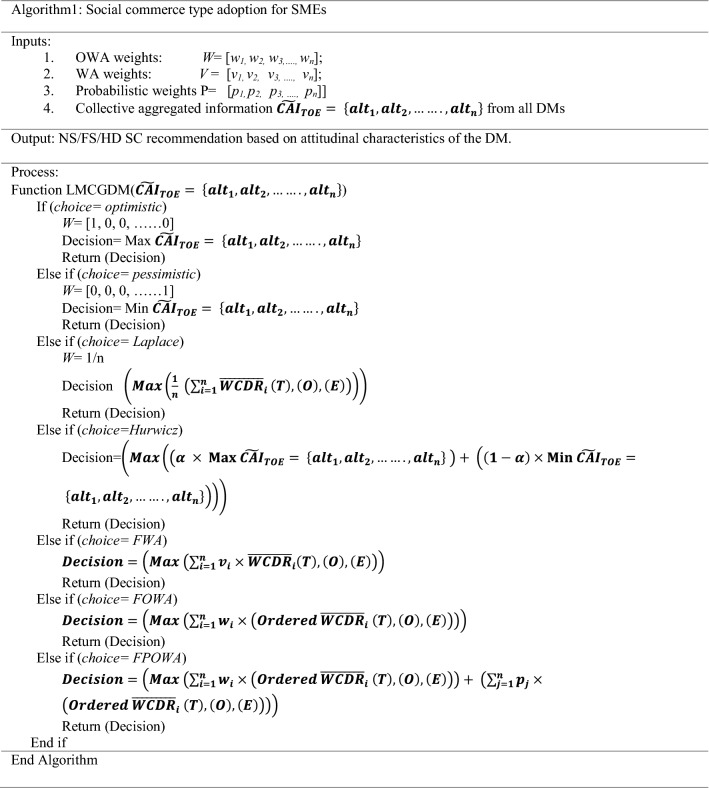


## Case Analysis

This section develops an illustrative example to demonstrate the working of the proposed approach in a linguistic multi-criteria group decision-making problem. In this case study, we analyse the decision making process under uncertainty in which the DM do not know what happens in future. We assessed the information with fuzzy triangular numbers. Different subjective methods using FAHP and FOWA are applied to demonstrate the decision-maker's attitudinal characteristics in a complex, uncertain environment.

In this study, we considered three SMEs with e-commerce websites and are willing to adopt social commerce for their business. The details of companies and experts are kept private to preserve confidentiality. All SMEs are Australian based and running their businesses for at least ten years. One of the companies is a clothing business situated in Dandenong South, a suburb in the southeast of Melbourne’s central business district. The second company is Sydney based auto parts company that sells products from various range of automotive brands. The third company is a food business that sells locally farmed fresh food. This business is situated in Greater Western Sydney. The distribution of employees in all three business companies is 36:71:38, working under the supervision of independent owners.

The decision-maker is divided into five groups based on their functional department. All DMs were invited to survey the inclusion of the following alternatives for their business.

Alternative 1: Onsite social commerce (NS) adoption.

Alternative 2: Offsite social commerce (FS) adoption.

Alternative 3: Hybrid social commerce (HD) adoption.

The role of the decision-makers are as follows:

DM1: is the company’s Owner or CEO.

DM2: is the Business Manager.

DM3: is the IT Manager.

DM4: is the Marketing Manager.

DM5: is the Financial Manager.

## Implementation

This section presents the implementation and of the proposed approach by considering above discussed scenario.

### Alternative evaluation by DMs

All DMs offer their opinions regarding the type of social commerce that best fit their business. The information is very imprecise. The DM analyses the information based on the fuzzy linguistic term presented in Table 4. Linguistic results are shown in Table 6.

**Table 6:** Linguistic alternative evaluation matrix

Criteria	Sub-criteria	Alternatives	DM1	DM2	DM3	DM4	DM5
Technology	Relative advantage	Onsite	VH	H	VH	H	VH
		Offsite	H	VH	H	H	VH
		Both	VH	VH	H	VH	VH
	Compatibility	Onsite	MH	MH	H	H	MH
		Offsite	H	H	VH	H	MH
		Both	MH	H	VH	H	MH
	Complexity	Onsite	VH	VH	H	VH	H
		Offsite	M	ML	L	L	ML
		Both	MH	MH	H	MH	H
	Security and privacy	Onsite	M	MH	M	ML	M
		Offsite	H	VH	H	VH	VH
		Both	MH	M	MH	M	M
	Reliability	Onsite	H	H	VH	VH	H
		Offsite	MH	MH	H	MH	M
		Both	MH	H	H	H	H
	Scalability	Onsite	L	ML	ML	M	L
		Offsite	VH	H	VH	H	VH
		Both	H	M	ML	L	M
Organization	Organization readiness	Onsite	M	H	M	M	M
		Offsite	VH	H	VH	H	VH
		Both	M	H	M	M	M
	Firm size	Onsite	VH	H	H	H	M
		Offsite	H	VH	VH	M	VH
		Both	H	VH	VH	M	M
	Top management support	Onsite	VH	H	H	M	H
		Offsite	M	H	H	H	H
		Both	M	M	VH	H	H
Environment	Competitive pressure	Onsite	H	VH	VH	H	VH
		Offsite	VH	H	H	VH	VH
		Both	VH	VH	VH	H	VH
	Trading partner pressure	Onsite	H	H	H	M	H
		Offsite	VH	VH	VH	VH	H
		Both	VH	VH	VH	H	VH
	Government regulations	Onsite	ML	M	L	ML	M
		Offsite	VH	VH	VH	H	VH
		Both	H	H	VH	H	VH

Each linguistic terms are converted to fuzzy numbers as presented in Table 7.

**Table 7:** Fuzzy number for decision-maker recommendation for the type of social commerce

Criteria	Sub-criteria	Alternatives	DM1	DM2	DM3	DM4	DM5
Technology	Relative advantage	Onsite	(90, 100, 100)	(70, 90, 100)	(90, 100, 100)	(70, 90, 100)	(90, 100, 100)
		Offsite	(70, 90, 100)	(90, 100, 100)	(70, 90, 100)	(70, 90, 100)	(90, 100, 100)
		Both	(90, 100, 100)	(90, 100, 100)	(70, 90, 100)	(90, 100, 100)	(90, 100, 100)
	Compatibility	Onsite	(50, 70, 90)	(50, 70, 90)	(70, 90, 100)	(70, 90, 100)	(50, 70, 90)
		Offsite	(70, 90, 100)	(70, 90, 100)	(90, 100, 100)	(70, 90, 100)	(50, 70, 90)
		Both	(50, 70, 90)	(70, 90, 100)	(90, 100, 100)	(70, 90, 100)	(50, 70, 90)
	Complexity	Onsite	(90, 100, 100)	(90, 100, 100)	(70, 90, 100)	(90, 100, 100)	(70, 90, 100)
		Offsite	(30, 50, 70)	(10, 30, 50)	(0, 10, 30)	(0, 10, 30)	(10, 30, 50)
		Both	(50, 70, 90)	(50, 70, 90)	(70, 90, 100)	(50, 70, 90)	(70, 90, 100)
	Security and privacy	Onsite	(10, 30, 50)	(50, 70, 90)	(30, 50, 70)	(10, 30, 50)	(30, 50, 70)
		Offsite	(70, 90, 100)	(90, 100, 100)	(70, 90, 100)	(90, 100, 100)	(90, 100, 100)
		Both	(50, 70, 90)	(30, 50, 70)	(50, 70, 90)	(30, 50, 70)	(30, 50, 70)
	Reliability	Onsite	(70, 90, 100)	(70, 90, 100)	(90, 100, 100)	(90, 100, 100)	(70, 90, 100)
		Offsite	(50, 70, 90)	(50, 70, 90)	(70, 90, 100)	(50, 70, 90)	(30, 50, 70)
		Both	(50, 70, 90)	(70, 90, 100)	(70, 90, 100)	(70, 90, 100)	(70, 90, 100)
	Scalability	Onsite	(0, 10, 30)	(10, 30, 50)	(10, 30, 50)	(30, 50, 70)	(00, 10, 30)
		Offsite	(90, 100, 100)	(70, 90, 100)	(90, 100, 100)	(70, 90, 100)	(90, 100, 100)
		Both	(70, 90, 100)	(30, 50, 70)	(10, 30, 50)	(0, 10, 30)	(30, 50, 70)
Organization	Organization readiness	Onsite	(30, 50, 70)	(70, 90, 100)	(30, 50, 70)	(30, 50, 70)	(30, 50, 70)
		Offsite	(90, 100, 100)	(70, 90, 100)	(90, 100, 100)	(70, 90, 100)	(90, 100, 100)
		Both	(30, 50, 70)	(70, 90, 100)	(30, 50, 70)	(30, 50, 70)	(30, 50, 70)
	Firm size	Onsite	(90, 100, 100)	(70, 90, 100)	(70, 90, 100)	(70, 90, 100)	(30, 50, 70)
		Offsite	(70, 90, 100)	(90, 100, 100)	(90, 100, 100)	(30, 50, 70)	(90, 100, 100)
		Both	(70, 90, 100)	(90, 100, 100)	(90, 100, 100)	(30, 50, 70)	(30, 50, 70)
	Top management support	Onsite	(90, 100, 100)	(70, 90, 100)	(70, 90, 100)	(30, 50, 70)	(70, 90, 100)
		Offsite	(30, 50, 70)	(70, 90, 100)	(70, 90, 100)	(70, 90, 100)	(70, 90, 100)
		Both	(30, 50, 70)	(30, 50, 70)	(90, 100, 100)	(70, 90, 100)	(70, 90, 100)
Environment	Competitive pressure	Onsite	(70, 90, 100)	(90, 100, 100)	(90, 100, 100)	(70, 90, 100)	(90, 100, 100)
		Offsite	(90, 100, 100)	(70, 90, 100)	(70, 90, 100)	(90, 100, 100)	(90, 100, 100)
		Both	(90, 100, 100)	(90, 100, 100)	(90, 100, 100)	(70, 90, 100)	(90, 100, 100)
	Trading partner pressure	Onsite	(70, 90, 100)	(70, 90, 100)	(70, 90, 100)	(30, 50, 70)	(70, 90, 100)
		Offsite	(90, 100, 100)	(90, 100, 100)	(90, 100, 100)	(90, 100, 100)	(70, 90, 100)
		Both	(90, 100, 100)	(90, 100, 100)	(90, 100, 100)	(70, 90, 100)	(90, 100, 100)
	Government regulations	Onsite	(10, 30, 50)	(30, 50, 70)	(0, 10, 30)	(10, 30, 50)	(30, 50, 70)
		Offsite	(90, 100, 100)	(90, 100, 100)	(90, 100, 100)	(70, 90, 100)	(90, 100, 100)
		Both	(70, 90, 100)	(70, 90, 100)	(90, 100, 100)	(70, 90, 100)	(90, 100, 100)

### The relative importance of criteria weights

To assess the relative weight of criteria, the DMs are asked to evaluate each criterion's relative importance using the scale of relative importance, as presented in Table 5. The pairwise comparison matrix using Eqs. (6–9) is shown in Table 8.

**Table 8:** Fuzzy pairwise evaluation matrix

	Technology	Organisation	Environment
Technology	(1, 1, 1)	(4, 5, 6)	(0.33, 0.50, 1)
Organisation	(0.17, 0.20, 0.25)	(1, 1, 1)	(0.14, 0.17, 0.2)
Environment	(1, 2, 3)	(5, 6, 7)	(1, 1, 1)

The comparison matrix is then normalised to get the criteria weights, as presented in Table 9.

**Table 9:** Criteria weight using FAHP

	Technology	Organisation	Environment	Fuzzy geometric mean value (${\tilde{r }}_{i}$)	Fuzzy weights (${\tilde{w }}_{i}$)	Crisp weights ${w}_{i}$	Normalised weight
Technology	(1, 1, 1)	(4, 5, 6)	(0.33, 0.50, 1)	(1.07, 1.26, 1.57)	(0.26, 0.35, 0.52)	0.38	0.37
Organisation	(0.17, 0.20, 0.25)	(1, 1, 1)	(0.14, 0.17 ,0.2)	(0.39, 0.43 ,0.47)	(0.09, 0.12, 0.16)	0.12	0.12
Environment	(1, 2, 3)	(5, 6, 7)	(1, 1, 1)	(1.49, 1.86, 2.14)	(0.36, 0.52, 0.71)	0.53	0.51

The consistency index and consistency ratio is determined as:

Consistency index (CI) = (3.12852 – 3) / 2 = 0.06426.

Consistency ratio (CR) = 0.114/ 1.25 = 0.05 < 0.10.

Therefore, we can say that our matrix is reasonably consistent, and we then move to a decision-making module.

The obtained relative importance shows that the Environment factor has a high weightage (0.36, 0.52, 0.71) of 51%. Technology have an importance (0.26, 0.35, 0.52) of 37% and Organisation have an importance (0.09, 0.12, 0.16) of 12%. Applying the weights of each criterion on DMs fuzzy recommendation, we get weighted criteria of DM recommendation as presented in Table 10.

**Table 10:** Applying relative criteria weights on decision-makers recommendations

Criteria	Sub-criteria	Alternatives	DM1	DM2	DM3	DM4	DM5
Technology (0.26, 0.35, 0.52)	Relative advantage	Onsite	(23.4, 35, 52)	(18.2, 31.5, 52)	(23.4, 35, 52)	(18.2, 31.5, 52)	(23.4, 35, 52)
		Offsite	(18.2, 31.5, 52)	(23.4, 35, 52)	(18.2, 31.5, 52)	(18.2, 31.5, 52)	(23.4, 35, 52)
		Both	(23.4, 35, 52)	(23.4, 35, 52)	(18.2, 31.5, 52)	(23.4, 35, 52)	(23.4, 35, 52)
	Compatibility	Onsite	(13, 24.5, 46.8)	(13, 24.5, 46.8)	(18.2, 31.5, 52)	(18.2, 31.5, 52)	(13, 24.5, 46.8)
		Offsite	(18.2, 31.5, 52)	(18.2, 31.5, 52)	(23.4, 35, 52)	(18.2, 31.5, 52)	(13, 24.5, 46.8)
		Both	(13, 24.5, 46.8)	(18.2, 31.5, 52)	(23.4, 35, 52)	(18.2, 31.5, 52)	(13, 24.5, 46.8)
	Complexity	Onsite	(23.4, 35, 52)	(23.4, 35, 52)	(18.2, 31.5, 52)	(23.4, 35, 52)	(18.2, 31.5, 52)
		Offsite	(7.8, 17.5, 36.4)	(2.6, 10.5, 26)	(0, 3.5, 15.6)	(0, 3.5, 15.6)	(2.6, 10.5, 26)
		Both	(13, 24.5, 46.8)	(13, 24.5, 46.8)	(18.2, 31.5, 52)	(13, 24.5, 46.8)	(18.2, 31.5, 52)
	Security and privacy	Onsite	(2.6, 10.5, 26)	(13, 24.5, 46.8)	(7.8, 17.5, 36.4)	(2.6, 10.5, 26)	(7.8, 17.5, 36.4)
		Offsite	(18.2, 31.5, 52)	(23.4, 35, 52)	(18.2, 31.5, 52)	(23.4, 35, 52)	(23.4, 35, 52)
		Both	(13, 24.5, 46.8)	(7.8, 17.5, 36.4)	(13, 24.5, 46.8)	(7.8, 17.5, 36.4)	(7.8, 17.5, 36.4)
	Reliability	Onsite	(18.2, 31.5, 52)	(18.2, 31.5, 52)	(23.4, 35, 52)	(23.4, 35, 52)	(18.2, 31.5, 52)
		Offsite	(13, 24.5, 46.8)	(13, 24.5, 46.8)	(18.2, 31.5, 52)	(13, 24.5, 46.8)	(7.8, 17.5, 36.4)
		Both	(13, 24.5, 46.8)	(18.2, 31.5, 52)	(18.2, 31.5, 52)	(18.2, 31.5, 52)	(18.2, 31.5, 52)
	Scalability	Onsite	(0, 3.5, 15.6)	(2.6, 10.5, 26)	(2.6, 10.5, 26)	(7.8, 17.5, 36.4)	(0, 3.5, 15.6)
		Offsite	(23.4, 35, 52)	(18.2, 31.5, 52)	(23.4, 35, 52)	(18.2, 31.5, 52)	(23.4, 35, 52)
		Both	(18.2, 31.5, 52)	(7.8, 17.5, 36.4)	(2.6, 10.5, 26)	(0, 3.5, 15.6)	(7.8, 17.5, 36.4)
Organization (0.09, 0.12, 0.16)	Organization readiness	Onsite	(2.7, 6, 11.2)	(6.3, 10.8, 16)	(2.7, 6, 11.2)	(2.7, 6, 11.2)	(2.7, 6, 11.2)
		Offsite	(8.1, 12, 16)	(6.3, 10.8, 16)	(8.1, 12, 16)	(6.3, 10.8, 16)	(8.1, 12, 16)
		Both	(2.7, 6, 11.2)	(6.3, 10.8, 16)	(2.7, 6, 11.2)	(2.7, 6, 11.2)	(2.7, 6, 11.2)
	Firm size	Onsite	(8.1, 12, 16)	(6.3, 10.8, 16)	(6.3, 10.8, 16)	(6.3, 10.8, 16)	(2.7, 6, 11.2)
		Offsite	(6.3, 10.8, 16)	(8.1, 12, 16)	(8.1, 12, 16)	(2.7, 6, 11.2)	(8.1, 12, 16)
		Both	(6.3, 10.8, 16)	(8.1, 12, 16)	(8.1, 12, 16)	(2.7, 6, 11.2)	(2.7, 6, 11.2)
	Top management support	Onsite	(8.1, 12, 16)	(6.3, 10.8, 16)	(6.3, 10.8, 16)	(2.7, 6, 11.2)	(6.3, 10.8, 16)
		Offsite	(2.7, 6, 11.2)	(6.3, 10.8, 16)	(6.3, 10.8, 16)	(6.3, 10.8, 16)	(6.3, 10.8, 16)
		Both	(2.7, 6, 11.2)	(2.7, 6, 11.2)	(8.1, 12, 16)	(6.3, 10.8, 16)	(6.3, 10.8, 16)
Environment (0.36, 0.52, 0.71)	Competitive pressure	Onsite	(25.2, 46.8, 71)	(32.4, 52, 71)	(32.4, 52, 71)	(25.2, 46.8, 71)	(32.4, 52, 71)
		Offsite	(32.4, 52, 71)	(25.2, 46.8, 71)	(25.2, 46.8, 71)	(32.4, 52, 71)	(32.4, 52, 71)
		Both	(32.4, 52, 71)	(32.4, 52, 71)	(32.4, 52, 71)	(25.2, 46.8, 71)	(32.4, 52, 71)
	Trading partner pressure	Onsite	(25.2, 46.8, 71)	(25.2, 46.8, 71)	(25.2, 46.8, 71)	(10.8, 26, 49.7)	(25.2, 46.8, 71)
		Offsite	(32.4, 52, 71)	(32.4, 52, 71)	(32.4, 52, 71)	(32.4, 52, 71)	(25.2, 46.8, 71)
		Both	(32.4, 52, 71)	(32.4, 52, 71)	(32.4, 52, 71)	(25.2, 46.8, 71)	(32.4, 52, 71)
	Government regulations	Onsite	(3.6, 15.6, 35.5)	(10.8, 26, 49.7)	(0, 5.2, 21.30)	(3.6, 15.6, 35.5)	(10.8, 26, 49.7)
		Offsite	(32.4, 52, 71)	(32.4, 52, 71)	(32.4, 52, 71)	(25.2, 46.8, 71)	(32.4, 52, 71)
		Both	(25.2, 46.8, 71)	(25.2, 46.8, 71)	(32.4, 52, 71)	(25.2, 46.8, 71)	(32.4, 52, 71)

### Importance of the decision-makers

Although all decision-makers evaluate each alternative, however, the decision made by all decision-makers are not equally important. A slight variation in the DMs' recommendation having higher importance than other DMs significantly changes the decision-making process. The study asked about the importance of DMs for this problem during the survey process to handle the situation. The organisation's owner or CEO has the highest importance than the other decision-makers, followed by a financial manager and IT managers having equal importance, then business managers and marketing managers. The weighting vectors that represents the weights of the DMs are as follows—DM1 = 0.35, DM5 = 0.25, DM2 = 0.15, DM3 = 0.15, DM4 = 0.10. By applying the relative weights of each DM in the decision-making process, we get the weighted DM recommendation for each alternative, as presented in Table 11.

**Table 11:** Applying relative importance of decision-makers

Criteria	Sub-criteria	Alternatives	DM1 = 0.35	DM2 = 0.15	DM3 = 0.15	DM4 = 0.10	DM5 = 0.25
Technology	Relative advantage	Onsite	(8.19,12.25,18.20)	(2.73, 4.73, 7.80)	(13.5, 15, 15)	(1.82, 3.15, 5.2)	(1.46, 2.19, 13)
		Offsite	(6.37,11.01,18.20)	(13.5, 15, 15)	(2.73, 4.73, 7.80)	(1.82, 3.15, 5.2)	(1.46, 2.19, 13)
		Both	(8.19,12.25,18.20)	(13.5, 15, 15)	(2.73, 4.73, 7.80)	(2.34, 3.5 5.2)	(1.46, 2.19, 13)
	Compatibility	Onsite	(4.55, 8.56, 16.38)	(1.95, 3.68, 7.02)	(2.73, 4.73, 7.80)	(1.82, 3.15, 5.2)	(12.5, 17.5, 22.5)
		Offsite	(6.37,11.01,18.20)	(2.73, 4.73, 7.80)	(13.5, 15, 15)	(1.82, 3.15, 5.2)	(12.5, 17.5, 22.5)
		Both	(4.55, 8.56, 16.38)	(2.73, 4.73, 7.80)	(13.5, 15, 15)	(1.82, 3.15, 5.2)	(12.5, 17.5, 22.5)
	Complexity	Onsite	(8.19,12.25,18.20)	(13.5, 15, 15)	(2.73, 4.73, 7.80)	(2.34, 3.5 5.2)	(4.55, 7.88, 13)
		Offsite	(2.73,6.13,12.74)	(0.39, 1.58, 3.90)	(0, 0.53, 2.34)	(0, 0.35, 1.56)	(0.65, 2.63, 6.50)
		Both	(4.55, 8.56, 16.38)	(1.95, 3.68, 7.02)	(2.73, 4.73, 7.80)	(1.30, 2.45, 4.68)	(4.55, 7.88, 13)
	Security and privacy	Onsite	(0.91, 3.68, 9.10)	(1.95, 3.68, 7.02)	(1.17, 2.63, 5.46)	(0.26, 1.05, 2.6)	(1.95, 4.38, 9.10)
		Offsite	(6.37,11.01,18.20)	(13.5, 15, 15)	(2.73, 4.73, 7.80)	(2.34, 3.5 5.2)	(1.46, 2.19, 13)
		Both	(4.55, 8.56, 16.38)	(1.17, 2.63, 5.46)	(1.95, 3.68, 7.02)	(0.78, 1.75, 3.64)	(1.95, 4.38, 9.10)
	Reliability	Onsite	(6.37,11.01,18.20)	(2.73, 4.73, 7.80)	(13.5, 15, 15)	(2.34, 3.5 5.2)	(4.55, 7.88, 13)
		Offsite	(4.55, 8.56, 16.38)	(1.95, 3.68, 7.02)	(2.73, 4.73, 7.80)	(1.30, 2.45, 4.68)	(1.95, 4.38, 9.10)
		Both	(4.55, 8.56, 16.38)	(2.73, 4.73, 7.80)	(2.73, 4.73, 7.80)	(1.82, 3.15, 5.2)	(4.55, 7.88, 13)
	Scalability	Onsite	(0, 1.23, 5.46)	(0.39, 1.58, 3.90)	(0.39, 1.58, 3.90)	(0.78, 1.75, 3.64)	(0, 0.86, 3.90)
		Offsite	(8.19,12.25,18.20)	(2.73, 4.73, 7.80)	(13.5, 15, 15)	(1.82, 3.15, 5.2)	(1.46, 2.19, 13)
		Both	(6.37,11.01,18.20)	(1.17, 2.63, 5.46)	(0.39, 1.58, 3.90)	(0, 3.5, 15.6)	(1.95, 4.38, 9.10)
Organization	Organization readiness	Onsite	(0.95, 2.10, 3.92)	(0.95, 1.62, 2.40)	(0.41, 0.90 1.68)	(0.27, 0.60, 1.12)	(0.68, 1.50, 2.80)
		Offsite	(2.84, 4.20, 5.60)	(0.95, 1.62, 2.40)	(1.22,1.80, 2.40)	(0.63, 1.08, 1.6)	(2.03, 3, 4)
		Both	(0.95, 2.10, 3.92)	(0.95, 1.62, 2.40)	(0.41, 0.90 1.68)	(0.27, 0.60, 1.12)	(0.68, 1.50, 2.80)
	Firm size	Onsite	(2.84, 4.20, 5.60)	(0.95, 1.62, 2.40)	(0.95, 1.62, 2.40)	(0.63, 1.08, 1.6)	(0.68, 1.50, 2.80)
		Offsite	(2.21, 3.78, 5.60)	(1.22, 1.80, 2.40)	(1.22,1.80, 2.40)	(0.27, 0.60, 1.12)	(2.03, 3, 4)
		Both	(2.21, 3.78, 5.60)	(1.22, 1.80, 2.40)	(1.22,1.80, 2.40)	(0.27, 0.60, 1.12)	(0.68, 1.50, 2.80)
	Top management support	Onsite	(2.84, 4.20, 5.60)	(0.95, 1.62, 2.40)	(0.95, 1.62, 2.40)	(0.27, 0.60, 1.12)	(1.58, 2.70, 4)
		Offsite	(0.95, 2.10, 3.92)	(0.95, 1.62, 2.40)	(0.95, 1.62, 2.40)	(0.63, 1.08, 1.6)	(1.58, 2.70, 4)
		Both	(0.95, 2.10, 3.92)	(0.41, 0.90, 1.68)	(1.22,1.80, 2.40)	(0.63, 1.08, 1.6)	(1.58, 2.70, 4)
Environment	Competitive pressure	Onsite	(8.82, 16.38, 24.85)	(13.5, 15, 15)	(4.86, 7.80, 10.65)	(2.52, 4.68, 7.1)	(8.10, 13, 17.75)
		Offsite	(11.34, 18.20, 24.85)	(3.78, 7.02, 10.65)	(3.78, 7.02, 10.65)	(3.24, 5.20, 7.10)	(8.10, 13, 17.75)
		Both	(11.34, 18.20, 24.85)	(13.5, 15, 15)	(4.86, 7.80, 10.65)	(2.52, 4.68, 7.1)	(8.10, 13, 17.75)
	Trading partner pressure	Onsite	(8.82, 16.38, 24.85)	(3.78, 7.02, 10.65)	(3.78, 7.02, 10.65)	(1.08, 2.60, 4.97)	(6.30, 11.70, 17.75)
		Offsite	(11.34, 18.20, 24.85)	(13.5, 15, 15)	(4.86, 7.80, 10.65)	(3.24, 5.20, 7.10)	(6.30, 11.70, 17.75)
		Both	(11.34, 18.20, 24.85)	(13.5, 15, 15)	(4.86, 7.80, 10.65)	(2.52, 4.68, 7.1)	(8.10, 13, 17.75)
	Government regulations	Onsite	(1.26, 5.46, 12.43)	(1.62, 3.90, 7.46)	(0, 0.78, 3.20)	(0.36, 1.56, 3.55)	(2.70, 6.50, 12.43)
		Offsite	(11.34, 18.20, 24.85)	(13.5, 15, 15)	(4.86, 7.80, 10.65)	(2.52, 4.68, 7.1)	(8.10, 13, 17.75)
		Both	(8.82, 16.38, 24.85)	(3.78, 7.02, 10.65)	(4.86, 7.80, 10.65)	(2.52, 4.68, 7.1)	(8.10, 13, 17.75)

### Collective DMs recommendation for TOE elements

The collective results for all DMs based on technology, organisation and element criteria is presented in Tables 12, 13, 14, and aggregated general expected results is shown in Table 15.

**Table 12:** Collective results for Technology

Criteria	Technology					
	Relative advantage	Compatibility	Complexity	Security and privacy	Reliability	Scalability
Onsite	(1.46, 7.46, 18.20)	(1.82, 7.52, 22.5)	(2.34,8.67,18.20)	(0.26, 3.08, 9.10)	(2.34, 8.42, 18.20)	(0, 1.4, 5.46)
Offsite	(1.46, 7.22,18.20)	(1.82,10.28,22.5)	(0, 2.28,12.74)	(1.46, 7.29,18.20)	(1.30, 4.76, 16.38)	(1.46, 7.46, 18.20)
Hybrid	(1.46,7.54,18.20)	(1.82, 9.79, 22.5)	(1.30, 5.46, 16.38)	(0.78, 4.2, 16.38)	(1.82, 5.81, 16.38)	(0, 4.62,18.20)

**Table 13:** Collective results for Organisation

Criteria	Organisation		
	Organization readiness	Firm size	Top management support
Onsite	(0.27, 1.34, 3.92)	(0.63, 2.00, 5.60)	(0.27, 2.15, 5.60)
Offsite	(0.63, 2.34, 5.60)	(0.27, 2.20, 5.60)	(0.63, 1.82, 4)
Hybrid	(0.27, 1.34, 3.92)	(0.27, 1.90, 5.60)	(0.41, 1.72, 4)

**Table 14:** Collective results for Environment

Criteria	Environment		
	Competitive pressure	Trading partner pressure	Government regulations
Onsite	(2.52, 11.37, 24.85)	(1.08, 8.94, 24.85)	(0, 3.64, 12.43)
Offsite	(3.24, 10.09, 24.85)	(3.24, 11.58, 24.85)	(2.52, 11.74, 24.85)
Hybrid	(2.52, 11.74, 24.85)	(2.52, 11.74, 24.85)	(2.52, 9.78, 24.85)

**Table 15:** Aggregated general expected results

	Technology	Organisation	Environment
Onsite	(1.37, 6.09, 15.28)	(0.39, 1.83, 5.04)	(1.2, 7.95, 20.71)
Offsite	(1.25, 6.55, 17.70)	(0.51, 2.12, 5.07)	(3, 11.14, 24.85)
Hybrid	(1.20, 6.24, 18.01)	(0.32, 1.65, 4.51)	(2.52, 11.09, 24.85)

### Decision-making process

To portray heterogeneous attitudinal characteristics of the decision makers, multiple aggregation methods are considered. Selected fuzzy aggregation method are – FMax, FMin, Laplace criteria, Hurwicz criteria, FWA, FOWA and FPOWA.

For the weighted average let us assume the weight vector WA = (0.45, 0.35, 0.20), for probabilistic weights P = (0.65, 0.25,0.10) and for the OWA weights: W = (0.25, 0.35, 0.40). The OWA weighting vector shows a bit pessimistic behaviour because higher weight is given to the end of the vector. Moreover, we further assume that the degree of optimism is 40% (α = 0.4), and a degree of pessimism is 60%. Table 16 presents the aggregated results.

**Table 16:** Aggregated results

	FMax	FMin	Laplace criteria	Hurwicz criteria	FWA	FOWA	FPOWA
Onsite (NS)	(1.37, 6.09, 15.28)	(0.39, 1.83, 5.04)	(0.99, 5.29, 13.68)	(0.79, 3.64, 9.13)	(1, 4.97, 12.78)	(0.92, 5.03, 13.09)	(1.81, 8.99, 23.02)
Offsite (FS)	(3, 11.14, 24.85)	(0.51, 2.12, 5.07)	(1.57, 6.60, 15.88)	(1.51, 5.73, 12.98)	(1.34, 5.92, 14.71)	(1.19, 5.93, 14.44)	(2, 10.19, 25.95)
Hybrid (HD)	(2.52, 11.09, 24.85)	(0.32, 1.65, 4.51)	(1.35, 6.33, 14.84)	(1.2, 5.43, 12.65)	(1.15, 5.61, 14.65)	(1.18, 5.61, 14.31)	(1.96, 9.62, 26.02)

**Fig. 4:**
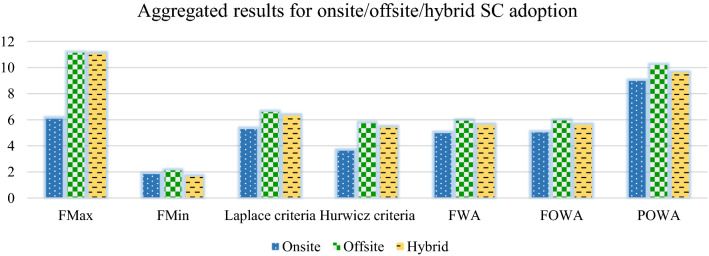
Aggregated result of all methods

To deepen the analysis results further, we present a graphical representation of aggregated results as presented in Fig. 4.

The bars in Fig. 4 presents the central value in FNs for each alternative. We see that for all methods, offsite adoption is the most preferred choice. Except for the FMin technique, the hybrid strategy is the second most preferred choice in all other approaches. The result indicates that if a company wants a social commerce feature on its website, it should keep offsite social commerce. To present the analysis results more comprehensively, Table 17 presents the ranking of alternatives for each decision-making method. The symbol ‘≺’ means precede in terms of preference.

**Table 17:** Ranking of alternatives

Approaches	Ordering
Fuzzy maximum	FS ≺ HD ≺ NS
Fuzzy minimum	FS ≺ OS ≺ HD
Laplace criteria	FS ≺ HD ≺ NS
Hurwicz criteria	FS ≺ HD ≺ NS
Fuzzy weighted average	FS ≺ HD ≺ NS
Fuzzy average	FS ≺ HD ≺ NS
Fuzzy ordered weighted average	FS ≺ HD ≺ NS

The optimal choice for SMEs is offsite social commerce adoption. The NS adoption requires an additional budget for more computing resources, additional personals with the required skills to build and maintain SC features, and an optimal security system to protect consumers' data against possible data breaches. Moreover, social media users' growth increases every day, and FS provides a broader audience, increases brand awareness, better communication, and effective interaction.

## Conclusion

Social commerce has revolutionised and reshaped the electronics business sector by facilitating consumers and service providers in various ways. With a limited budget, resources, and workforces, the SME also fails to adopt an optimal social commerce type. Different service providers have different attitudinal behaviour to take a possible risk of technology adoption. A single recommendation is not practicable for all attitudinal behaviour. This paper developed a novel MCGDM framework that have used TOE decision factors, FAHP and the OWA operator for decision recommendations. The proposed approach used FNs to deal with imprecise information under uncertain environments. The study considered a case study by considering three Australian-based SMEs to demonstrate the proposed approach's applicability. The analysis results demonstrated that the proposed approach handle complex, uncertain nonlinear problems in an effective way. In our future work, we will extend the proposed approach by considering other families of the linguistic OWA operator under uncertainty. Moreover, we will study the proposed framework's applicability in other areas, including cloud, IoT and economics.
